# Characteristics and outcomes of pregnant women hospitalized with severe maternal outcomes in eastern Ethiopia: Results from the Ethiopian Obstetric Surveillance System study

**DOI:** 10.1002/ijgo.15240

**Published:** 2023-11-14

**Authors:** Abera Kenay Tura, Marian Knight, Sagni Girma, Redwan Ahmed, Mohammed Yuya, Delayehu Bekele, Tahir Ahmed Hassen, Jelle Stekelenburg, Thomas van den Akker

**Affiliations:** ^1^ School of Nursing and Midwifery, College of Health and Medical Sciences Haramaya University Harar Ethiopia; ^2^ Department of Obstetrics and Gynecology University Medical Center Groningen, University of Groningen Groningen The Netherlands; ^3^ National Perinatal Epidemiology Unit University of Oxford Oxford UK; ^4^ Department of Obstetrics and Gynecology Leiden University Medical Center Leiden The Netherlands; ^5^ Department of Obstetrics and Gynecology Hiwot Fana Specialized University Hospital Harar Ethiopia; ^6^ School of Public Health, College of Health and Medical Sciences, Haramaya University Harar Ethiopia; ^7^ Department of Obstetrics and Gynecology St. Paul's Hospital Millennium Medical College Addis Ababa Ethiopia; ^8^ Center for Women's Health Research University of Newcastle Newcastle New South Wales Australia; ^9^ Department of Health Sciences, Global Health University Medical Center Groningen, University of Groningen The Netherlands; ^10^ Department of Obstetrics and Gynecology Leeuwarden Medical Center Leeuwarden The Netherlands; ^11^ Athena Institute, Vrije Universiteit Amsterdam Amsterdam The Netherlands

**Keywords:** audit, Ethiopia, maternal mortality, severe maternal morbidity, surveillance

## Abstract

**Objective:**

The aim of the present study was to identify facility‐based incidence of severe obstetric complications through a newly established obstetric surveillance system in eastern Ethiopia.

**Methods:**

Monthly registration of obstetric hemorrhage, eclampsia, uterine rupture, severe anemia and sepsis was introduced in 13 maternity units in eastern Ethiopia. At each hospital, a designated clinician reported details of women admitted during pregnancy, childbirth or within 42 days of termination of pregnancy from April 01, 2021 to March 31, 2022 developing any of these conditions. Detailed data on sociodemographic characteristics, obstetric complications and status at discharge were collected by trained research assistants.

**Results:**

Among 38 782 maternities during the study period, 2043 (5.3%) women had any of the five conditions. Seventy women died, representing a case fatality rate of 3.4%. The three leading reasons for admission were obstetric hemorrhage (972; 47.6%), severe anemia (727; 35.6%), and eclampsia (438; 21.4%). The majority of the maternal deaths were from obstetric hemorrhage (27/70; 38.6%) followed by eclampsia (17/70; 24.3%).

**Conclusion:**

Obstetric hemorrhage, severe anemia and eclampsia were the leading causes of severe obstetric complications in eastern Ethiopia. Almost one in 29 women admitted with obstetric complications died. Audit of quality of care is indicated to design tailored interventions to improve maternal survival and obstetric complications.

## Synopsis

One in 20 pregnant women admitted to hospitals in eastern Ethiopia developed one of the five major adverse obstetric conditions.

## 1 INTRODUCTION

A recent report from the WHO and other UN agencies estimated a maternal mortality ratio (MMR) of 267 maternal deaths per 100 000 live births in Ethiopia.^1^ Although this shows an overall reduction by 33.4% since 2017, compared to the stagnating global level,^2^ this is the fourth highest MMR after Nigeria, India, and the Democratic Republic of the Congo.^3^ Two‐thirds of maternal deaths result from hemorrhage, hypertensive disorders of pregnancy and sepsis.^4^ The remaining deaths are often associated with severe anemia, in the presence of other conditions such as hemorrhage. In addition, for every maternal death, 5 to 30 other women experience severe obstetric complications, but survive.^5^, ^6^ Reducing maternal mortality and severe maternal morbidity requires an understanding of the chain of events among women who died or survived severe obstetric complications for designing tailored interventions.^7^

Despite having one of the highest frequencies of maternal mortality and, by extension, severe maternal morbidity, there is no registration system for capturing such events in Ethiopia. As such, the majority of evidence comes from estimates based on models from the WHO or other agencies, periodic surveys such as demographic and health surveys, and emergency obstetric and newborn assessments.^1^, ^8^, ^9^, ^10^ A system for identification and review of maternal deaths to institute tailored response, called maternal and perinatal death surveillance and response (MPDSR), was started in Ethiopia in 2013 as per a WHO recommendation.^11^, ^12^ While MPDSR has become one of the sources of information for maternal (and later perinatal) mortality, the system was found to capture less than 10% of the expected deaths and its implementation suffered from problems related to a blame culture.^13^, ^14^, ^15^, ^16^

In many settings, registration of maternal mortality is undertaken as part of vital registration or in specific registries.^17^ Despite some underreporting, many high‐resource settings generate essential evidence from such registries.^18^ With the emerging concept of severe maternal morbidity, coupled with the low absolute number of maternal deaths, registration of rare disorders of pregnancy has been started in several high‐income countries in the last two decades.^19^ The UK Obstetric Surveillance System (UKOSS) has been the pioneer in establishing a system for monitoring rare disorders of pregnancy in all consultant led maternity units in the UK. Since its establishment in 2005, in addition to generating evidence resulting in change in guidelines or introduction of new ones, UKOSS has been a model for similar systems in several countries.^19^ Currently, similar obstetric surveillance systems have been established in the Netherlands, Italy, the Nordic countries, Australia, Slovakia, and Belgium, among others, collaborating under the International Network of Obstetric Survey Systems (INOSS).^20^ More recently, a feasibility study on establishing Canadian Obstetric Surveillance System is under way.^21^ Until recently, no low‐ and middle‐income countries had such systems until the piloting program in Assam State in India (IndOSS‐Assam), and its recent expansion to other Indian states (MaatHRI).^22^, ^23^

As part of introducing a nationwide system for monitoring obstetric conditions in Ethiopia, the pilot of the Ethiopian Obstetric Surveillance System (EthOSS) was initiated in eastern Ethiopia in April 2021.^24^ In this study, we present the first results of the EthOSS project by describing the regional facility‐based incidence of the priority conditions (obstetric hemorrhage, eclampsia, uterine rupture, sepsis, and severe anemia) in the surveillance system, as well as maternal characteristics of women who experienced these conditions in order to inform policy aiming to improve maternity care.

## 2 MATERIALS AND METHODS

This was a multicenter study conducted from April 01, 2021 to March 31, 2022.^24^ The Ethiopian Obstetric Surveillance System (EthOSS) is a regional system established to investigate a range of major obstetric conditions in Ethiopia. The study was established through adapting the UK Obstetric Surveillance System (UKOSS) and NethOSS (Netherlands Obstetric Surveillance System) methodologies to the Ethiopian context.^19^, ^25^ Adaptation of the UKOSS methodology to the Ethiopian context and details of the EthOSS methodology have been described elsewhere.^24^ In brief, a designated midwife monthly reported on the number of cases from selected major obstetric conditions—obstetric hemorrhage, eclampsia, uterine rupture, severe anemia, and sepsis—and maternal deaths in their respective maternity units. On receiving the reports, EthOSS dispatched data collectors for verification of the eligibility of reported conditions and to collect detailed information about the woman and related perinatal outcomes.

The EthOSS project was a prospective multicenter facility‐based cohort study. All women admitted in public hospitals in eastern Ethiopia—Harari Region, Dire Dawa City Administration, East Hararghe and West Hararghe zones—constituted the source population whereas women with obstetric hemorrhage, eclampsia, uterine rupture, sepsis, and severe anemia were the study population. These conditions were identified and selected through review of the literature and considering their potential for improving care.^4^, ^5^, ^13^, ^14^, ^26^, ^27^, ^28^, ^29^ All public hospitals in the study settings were invited to join the study and assign a senior obstetrician or emergency surgical officer and a midwife for coordinating the study. At the end of each month, the designated midwife reported the number of cases from the selected conditions and maternal deaths.

As described elsewhere, major adverse obstetric conditions—obstetric hemorrhage, eclampsia, uterine rupture, sepsis, and severe anemia—were selected for inclusion in the surveillance system by the EthOSS steering committee.^24^ Obstetric hemorrhage was defined as excessive bleeding (usually related to pregnancy) in a parturient. The definition included both antepartum and postpartum hemorrhage. Antepartum hemorrhage included severe bleeding from or into the genital tract, occurring from 28 + 0 weeks of pregnancy and prior to the birth of the baby while postpartum hemorrhage refers to excessive bleeding (more than 500 mL for vaginal delivery and 1000 mL for cesarean delivery) following the birth of a baby. Eclampsia was defined as diastolic blood pressure ≥90 mm Hg or proteinuria +3 and presence of convulsions or coma. Similarly, uterine rupture was defined as complete rupture of the uterus during labour, confirmed by laparotomy or autopsy. Sepsis was defined as a clinical suspicion of infection and three of the following: temperature >38°C, respiration rate <20/min, pulse rate >90/min, or WBC >12 000. Severe anemia was defined by a hemoglobin level of <7 mg/dL.^30^ The records of each woman who had been reported were reviewed for eligibility before collecting detailed information.

The EthOSS study was approved by the Institutional Health Research Ethics Review Committee (IHRERC) of the College of Health and Medical Sciences, Haramaya University, Ethiopia (ref no. IHRERC/024/2021); and the University of Oxford's Tropical Research Ethics Committee (OxTREC reference 530‐21). Informed consent and approval for the study was obtained from the administrators of each hospital. As there was no interview with women, the need for individual consent was waived.

Data were collected using KoboToolbox, an open‐source suite of tools for data collection and analysis. All collected data were checked for completeness and were exported to Stata 13 (StataCorp LP, College Station, TX, USA) for analysis. We used descriptive statistics, reporting means with standard deviations for continuous variables, and frequency and percentages for categorical variables. Differences between women who survived and died were compared using *x*2 tests and a *P* value less than 0.05 was used as a cutoff point for statistical significance.

## 3 RESULTS

All 13 hospitals in eastern Ethiopia reported on the selected conditions on a monthly basis followed by checking for eligibility and data collection by the EthOSS data collectors. Over the one‐year period, from a total of 38 782 maternities, 34 090 live births and 2043 women with any of the five conditions (including 70 maternal deaths) were registered. This corresponds to an MMR of 205 per 100 000 livebirths. The mean age of participants was 25.7 (±5.8) years, with the majority of them being 20–35 years old (1708; 84.0%) (Table 1).

**TABLE 1:** Sociodemographic characteristics of pregnant women with specific severe complications in maternity units in eastern Ethiopia (*n* = 2043).

Variable	Frequency	Percentage
Age (*n* = 2033)		
<20	249	12.3
20–35	1708	84.0
>35	76	3.7
Parity (*n* = 1333)		
1	21	1.6
2–4	233	17.5
>4	728 351	54.6 26.3
Admitted to the intensive care unit? (*n* = 2031)		
Yes	101	5
No	1930	95
Booked for antenatal care (*n* = 2012)		
Yes	611	30.4
No	1401	69.6
Number of ANC visits (*n* = 611)		
1	161	26.4
2–3	331	54.2
≥4	56	9.1
Missing	63	10.3
Maternal vital status at discharge		
Alive	1973	96.6
Died	70	3.4

Abbreviation: ANC, antenatal care.

**FIGURE 1:**
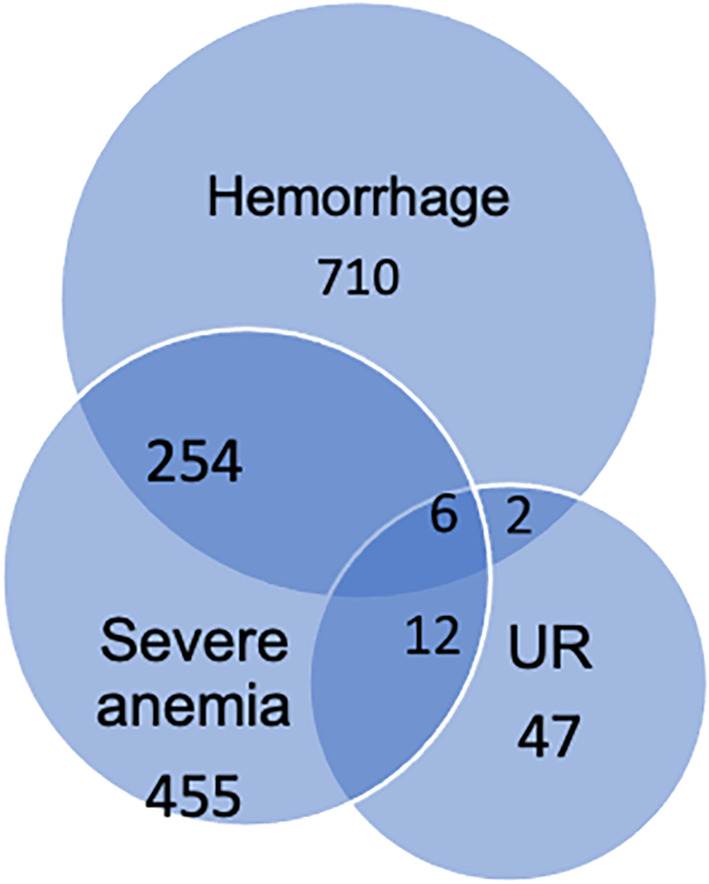
Concomitant occurrence of obstetric hemorrhage, severe anemia and uterine rupture among women admitted in eastern Ethiopia (*n* = 2043). Abbreviation: UR, uterine rupture.

By the time of discharge, 972 (47.6%), 727 (35.6%), and 438 (21.4%), of the women sustained obstetric hemorrhage, severe anemia, and eclampsia, respectively. More than half (56.1%) of the women were referred from lower facilities. Most women had given birth by the time of discharge (93.2%) (Table 2). It is important to note that some women experienced more than one condition. For example, 260 (35.8%) of women with severe anemia also had concomitant obstetric hemorrhage (Figure 1).

**TABLE 2:** Maternal and perinatal outcomes among pregnant women admitted with the five obstetric conditions in eastern Ethiopia (*n* = 2043).

Variable	Frequency	Percentage	Incidence per 1000 births^a^
EthOSS condition^b^			
Obstetric hemorrhage	972	47.6	25.1
Severe anemia	727	35.6	18.7
Eclampsia	438	21.4	11.3
Sepsis	215	10.5	5.5
Uterine rupture	67	3.3	1.7
Referral status (*n* = 2040)			
Referred from other facilities	1145	56.1	29.5
Not referred	895	43.9	23.1
Delivery status at discharge			
Gave birth or terminated pregnancy	1905	93.2	49.1
Still pregnant	16	0.8	0.4
Missing	122	6.0	3.1
Place of birth			
Health facility	1722	84.3	44.4
Home	260	12.7	6.7
Discharged pregnant	16	0.8	0.4
Missing	45	2.2	1.2
Fetal vital status at birth (*n* = 1905)			
Alive	1509	79.2	38.9
Stillborn	396	20.8	10.2
Neonatal vital status at discharge (*n* = 1509)			
Alive	1342	88.9	34.6
Died	21	1.4	0.5
Missing	146	9.7	3.8

^a^ Total number of births during the study period was 38 782.

^b^ Percentages exceed 100% since some women had more than one EthOSS condition.

Of the 2043 women with one of the five conditions, 101 (4.9%) were admitted to the intensive care unit and 70 (3.4%) died. Regarding perinatal outcomes, of the 1905 women who had given birth at the time of discharge, 396 (20.8%) had a stillbirth. Of the 1509 live born neonates for whom status at discharge was known, 21 (1.4%) had died prior to discharge (Table 2).

Compared to women who survived complications, women who died were more likely to be referred from lower facilities, admitted to an intensive care unit, and to have given birth by cesarean section. No difference was observed with regard to parity and antenatal booking status (Table 3).

**TABLE 3:** Sociodemographic and obstetric characteristics of women who died and survived complications (*n* = 2043).

Variable	Total, *n* (%)	Died, *n* (%)	Survived, *n* (%)	*P* value*
Age (*n* = 2033)				0.23
<20	249 (12.3)	4 (5.7)	245 (12.5)	
20–35	1708 (84.0)	63 (90%)	1645 (83.8)	
>35	76 (3.7)	3 (4.3)	73 (3.7)	
Referred from lower‐level facilities (*n* = 2040)				**<0.001**
Yes	1145 (56.1)	53 (77.9)	1092 (55.4)	
No	895 (43.9)	15 (22.1)	880 (44.6)	
Parity (*n* = 1333)				0.837
1	254 (19.1)	10 (19.2)	244 (19.0)	
2–4	588 (44.1)	21 (40.4)	567 (44.3)	
>4	491 (36.8)	21 (40.4)	470 (36.7)	
Admitted to the intensive care unit (*n* = 2031)				**<0.001**
Yes	101 (5)	13 (19.4)	88 (4.5)	
No	1930 (95)	54 (80.6)	1876 (95.5)	
Booked for antenatal care (*n* = 2012)				0.794
Yes	611 (30.4)	21 (31.8)	590 (30.3)	
No	1401 (69.6)	45 (68.2)	1356 (69.7)	
Mode of birth (*n* = 1701)				**0.024**
Cesarean section	383 (22.5)	19 (35.2)	364 (27.6)	
Vaginal	1318 (77.5)	35 (64.8)	1283 (72.4)	

* Chi‐square test.

Bold values indicates Referred from lower facilities *P* = 0.000227 and Admitted to the intensive care unit *P* = 0.00001.

## 4 DISCUSSION

The present study is the first of its kind to introduce a surveillance system for severe obstetric complications in Ethiopia, inspired by the experience of UKOSS and similar surveillance systems.^19^ After successfully introducing the EthOSS project in 13 public hospitals in eastern Ethiopia,^24^ we found an institutional MMR of 205 per 100 000 livebirths and a case fatality rate of 3.4% from five specific conditions. To the best of our knowledge, EthOSS is the first adaptation of an obstetric surveillance system in a low‐resource setting, Ethiopia, next to India.^23^ Despite becoming a common practice in many high‐income settings,^20^ our study revealed that it is feasible to implement an obstetric surveillance system in low resource settings with a high burden of maternal mortality and severe maternal morbidity.

In our study, the majority of the women reported from the participating hospitals had obstetric hemorrhage (47.6%) followed by severe anemia (35.6%). These findings are in accordance with our previous study that involved two of the 13 EthOSS participating hospitals.^6^ As might be anticipated, more than one third of the cases of severe anemia were associated with obstetric hemorrhage. Anemia might be a cause or consequence of hemorrhage, noting that the presence of anemia doubles the adverse consequences of hemorrhage.^31^ For example, the IndOSS‐Assam study (India) found a 50% increased risk of postpartum hemorrhage among women with moderate anemia and a 10‐fold increase among those with severe anemia.^32^ Although the exact mechanism by which anemia may cause postpartum hemorrhage is unclear, it could be related to a higher risk of uterine atony among anemic women as a result of impaired oxygen supply to the uterus. Moreover, consequences of postpartum hemorrhage might be more severe if hemoglobin was already low.^33^, ^34^

Hypertensive disorders of pregnancy are also among the leading causes of maternal deaths and morbidity. In our study, the incidence of eclampsia was found to be 11.3 per 1000 births, which is comparable to a finding reported in the national emergency obstetric and newborn 2016 assessment (10.5% had eclampsia among women who died).^35^ The current figure, however, is lower than the one reported in a previous study conducted in the region (30.7 per 1000 livebirths).^6^ The observed discrepancy might be attributed to the difference in the study population, that is, the current study included women from all levels of hospital, including primary level hospitals in rural areas, while the previous study was in urban areas only and included a tertiary university hospital.

In a country with one of the highest maternal mortality and morbidity ratios in the world, timely surveillance and evaluation of severe obstetric complications is crucial. We believe that the EthOSS platform can be used for surveillance of any adverse obstetric conditions in eastern Ethiopia and beyond. Such platforms have been found to be effective in establishing surveillance during the covid‐19 pandemic rapidly in the UK.^36^, ^37^ The EthOSS platform, and associated national scale up, could be essential for monitoring progress towards the 2030 Sustainable Development Goals (SDG) through provision of comprehensive data on maternal and perinatal outcomes. Unlike the existing platforms such as the maternal death surveillance and response—which largely focus on deaths alone—this platform can be used to provide denominator information since women who survived are also included. Our use of anonymous records and inclusion of both maternal deaths and severe maternal morbidity in the surveillance will also minimize the fear of blame which is becoming a major cause for underreporting in MPDSR.^15^, ^16^

Although we have successfully implemented EthOSS in all hospitals with active maternity units in eastern Ethiopia, our surveillance does not reflect population‐based estimates. Given that almost half of the women in this region still give birth at home, it is likely that women with severe complications are overrepresented in this facility‐based cohort, as compared to the general population.^38^

Unlike UKOSS and other obstetric surveillance programs from high‐income settings which collect anonymized data from hospital records, we used EthOSS data collectors for collecting detailed information after receiving case notification from the designated clinicians. This adaptation was required for the following reasons. First, since there is no electronic medical record system, data retrieval might be beyond the capacity of already busy clinicians in each hospital. Second, because of the often “incomplete” paper‐based records, there is a need to triangulate data from the medical records with information from the birth register and other registers. As a solution for the incompleteness of paper records, we are introducing an “EthOSS register and template” to be completed by managing clinicians. The template, to be part of a woman's medical records in the future, will be promoted for use by all managing clinicians and will be part of a quality audit for improving documentation. The template will contain essential sociodemographic and clinical information about women treated in all EthOSS hospitals. Moreover, it will enable us to trace referred women within the hospitals who will be uniquely identified minimizing double counting.

In conclusion, we found that that one in 20 (2043/38782) women who gave birth in the participating hospitals experienced one of the five conditions and one in 29 (70/2043) of those with any of the conditions died, indicating the importance of surveillance for adequately monitoring progress towards the 2030 targets in Ethiopia. Findings from our confidential enquiry into maternal deaths will be described in a subsequent study. Given the high burden of severe maternal morbidity and mortality, we are initiating quality improvement programs based on low‐dose high‐frequency training and context specific clinical guidance through adapting the PartoMa context‐specific approach to Ethiopia.^39^ Such quality improvement coupled with the EthOSS program will help us to monitor rates of adverse obstetric conditions and the effect of our interventions in the long run.

## AUTHOR CONTRIBUTIONS

Abera Kenay Tura, Marian Knight, and Thomas van den Akker designed the study and the analysis. Abera Kenay Tura, Sagni Girma, Redwan Ahmed, and Mohammed Yuya supervised the data collection. Abera Kenay Tura and Sagni Girma were project managers. Abera Kenay Tura analyzed the data and drafted the manuscript with support from Thomas van den Akker and Marian Knight. Marian Knight, Sagni Girma, Redwan Ahmed, Mohammed Yuya, Delayehu Bekele, Tahir Ahmed Hassen, Jelle Stekelenburg, and Thomas van den Akker reviewed the article. All authors read and approved the manuscript for submission.

## FUNDING INFORMATION

The study was funded by MRC (MR/T037962/1) as part of the 2019 Global Maternal and Neonatal Health Funding call. MK is a National Institute for Health and Care Research (NIHR) Senior Investigator. The views expressed are those of the authors and not necessarily those of the NHS, the MRC, the NIHR or the Department of Health and Social Care. The funders have no role in the study design, data collection and analysis, manuscript preparation or the decision for publication.

## CONFLICT OF INTEREST STATEMENT

None.

## Data Availability

Research data are not shared.
